# (Acetyl­acetone 3-hy­droxy-2-naphtho­yl­hydrazonato-κ^3^ *O*,*N*′,*O*′)(methoxo-κ*O*)oxidovanadate(V)

**DOI:** 10.1107/S1600536810028898

**Published:** 2010-07-24

**Authors:** Hon Wee Wong, Kong Mun Lo, Seik Weng Ng

**Affiliations:** a Department of Chemistry, University of Malaya, 50603 Kuala Lumpur, Malaysia

## Abstract

The tridentate Schiff base ligand in the title compound, [V(C_16_H_14_N_2_O_3_)(CH_3_O)O], has its O, N and O′ atoms spanning three basal positions of the square-based-pyrimidally V^V^ atom. The fourth basal site is occupied by a methoxo ligand, which results from a deprotonated methanol solvent molecule, and the oxido ligand occupies the apical position. The hy­droxy H atom forms an intra­molecular O—H⋯N hydrogen bond with an N-atom acceptor site.

## Related literature

For related vanadium(V) structures, see: Shao *et al.* (1988 ▶). The reaction of oxovanadium(IV) bis­(acetyl­acetonate), VO(acac)_2_, with aroylhydrazines in methanol yields Schiff-base complexes having the dinuclear [V(=O)(*μ*-OMe)_2_V(=O)]^4+^ core, see: Sarkari & Pal (2009 ▶).
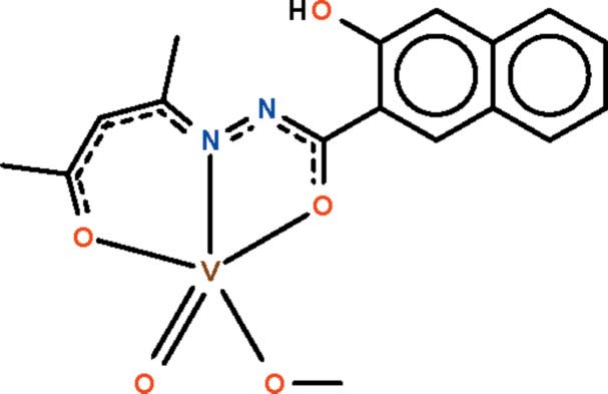


## Experimental

### 

#### Crystal data

[V(C_16_H_14_N_2_O_3_)(CH_3_O)O]
*M* *_r_* = 380.27
Triclinic, 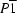

*a* = 7.7379 (8) Å
*b* = 9.7753 (10) Å
*c* = 11.4411 (11) Å
α = 86.618 (1)°
β = 70.821 (1)°
γ = 85.974 (1)°
*V* = 814.77 (14) Å^3^
*Z* = 2
Mo *K*α radiation
μ = 0.64 mm^−1^
*T* = 100 K
0.30 × 0.10 × 0.10 mm

#### Data collection

Bruker SMART APEX diffractometer
Absorption correction: multi-scan (*SADABS*; Sheldrick, 1996 ▶) *T*_min_ = 0.831, *T*_max_ = 0.939
7840 measured reflections
3716 independent reflections
3013 reflections with *I* > 2σ(*I*)
*R*_int_ = 0.027

#### Refinement

*R*[*F* ^2^ > 2σ(*F* ^2^)] = 0.038
*wR*(*F* ^2^) = 0.102
*S* = 1.03
3716 reflections
233 parameters
1 restraint
H atoms treated by a mixture of independent and constrained refinement
Δρ_max_ = 0.75 e Å^−3^
Δρ_min_ = −0.42 e Å^−3^

### 

Data collection: *APEX2* (Bruker, 2009 ▶); cell refinement: *SAINT* (Bruker, 2009 ▶); data reduction: *SAINT*; program(s) used to solve structure: *SHELXS97* (Sheldrick, 2008 ▶); program(s) used to refine structure: *SHELXL97* (Sheldrick, 2008 ▶); molecular graphics: *X-SEED* (Barbour, 2001 ▶); software used to prepare material for publication: *publCIF* (Westrip, 2010 ▶).

## Supplementary Material

Crystal structure: contains datablocks global, I. DOI: 10.1107/S1600536810028898/nk2050sup1.cif

Structure factors: contains datablocks I. DOI: 10.1107/S1600536810028898/nk2050Isup2.hkl

Additional supplementary materials:  crystallographic information; 3D view; checkCIF report

## Figures and Tables

**Table 1:** Hydrogen-bond geometry (Å, °)

*D*—H⋯*A*	*D*—H	H⋯*A*	*D*⋯*A*	*D*—H⋯*A*
O3—H3⋯N2	0.83 (1)	1.91 (2)	2.623 (2)	144 (3)

**Fig. 1.**
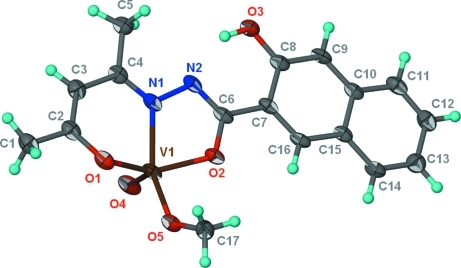
Displacement ellipsoid plot of VO(CH3O)(C16H14N2O3) at the 70% probability level; hydrogen atoms are drawn as spheres of arbitrary radius.

## supplementary crystallographic information

### Comment

The reaction of oxovanadium(IV) bis(acetylacetonate), VO(acac)_2_, with aroylhydrazines in acetonitrile yields vanadium(V) compounds of the formulation V_2_O_3_*L*_2_ (where *L* represents the doubly-deprotonated Schiff base). In methanol, the reaction yields Schiff-base complexes having the dinuclear [V(=O)(*µ*-OMe)_2_V(=O)]^4+^ core (Sarkari & Pal, 2009). In the present study, the reaction with a hydroxy-substituted naphthanoic acid hydrazide yields the expected vanadium(V) complex of the doubly-deprotonated Schiff base but a molecule of the solvent is reduced to a methoxide ion (Scheme I). The metal center has square-based pyramidal coordination geometry, with the *O*,*N*,*O*'-atoms of the Schiff base spanning the basal sites, the fourth basal site is occupied by a methoxy ligand and the apical site is occupied by an oxo ligand.

### Experimental

Bis(acetylacetonato)oxovanadium(IV) (0.20 g, 0.75 mmol) and 3-hydroxy-2-naphthoyl hydrazide (0.15 g, 0.75 mmol) were heated in methanol (100 ml) for one hour. The brown solution was filtered; slow evaporation of the filtrate afforded brown crystals.

### Refinement

C-bound H-atoms were placed in calculated positions (C—H 0.95 to 0.98 Å) and were included in the refinement in the riding model approximation, with *U*(H) set to 1.2 to 1.5*U*(C). The hydroxyl H-atom was located in a difference Fourier map, and was refined with a distance restraint of O–H 0.84 (1) Å; its isotropic displacement parameter was freely refined. There is no residual electron density near the methoxido O-atom.

### Crystal data

**Table:**

[V(C_16_H_14_N_2_O_3_)(CH_3_O)O]	*Z* = 2
*M**_r_* = 380.27	*F*(000) = 392
Triclinic, *P*1	*D*_x_ = 1.550 Mg m^−^^3^
Hall symbol: -P 1	Mo *K*α radiation, λ = 0.71073 Å
*a* = 7.7379 (8) Å	Cell parameters from 2783 reflections
*b* = 9.7753 (10) Å	θ = 2.8–28.1°
*c* = 11.4411 (11) Å	µ = 0.64 mm^−^^1^
α = 86.618 (1)°	*T* = 100 K
β = 70.821 (1)°	Prism, brown
γ = 85.974 (1)°	0.30 × 0.10 × 0.10 mm
*V* = 814.77 (14) Å^3^	

### Data collection

**Table:**

Bruker SMART APEX diffractometer	3716 independent reflections
Radiation source: fine-focus sealed tube	3013 reflections with *I* > 2σ(*I*)
graphite	*R*_int_ = 0.027
ω scans	θ_max_ = 27.5°, θ_min_ = 1.9°
Absorption correction: multi-scan (*SADABS*; Sheldrick, 1996)	*h* = −9→10
*T*_min_ = 0.831, *T*_max_ = 0.939	*k* = −12→12
7840 measured reflections	*l* = −14→14

### Refinement

**Table:**

Refinement on *F*^2^	Primary atom site location: structure-invariant direct methods
Least-squares matrix: full	Secondary atom site location: difference Fourier map
*R*[*F*^2^ > 2σ(*F*^2^)] = 0.038	Hydrogen site location: inferred from neighbouring sites
*wR*(*F*^2^) = 0.102	H atoms treated by a mixture of independent and constrained refinement
*S* = 1.02	*w* = 1/[σ^2^(*F*_o_^2^) + (0.0499*P*)^2^ + 0.2795*P*] where *P* = (*F*_o_^2^ + 2*F*_c_^2^)/3
3716 reflections	(Δ/σ)_max_ = 0.001
233 parameters	Δρ_max_ = 0.75 e Å^−^^3^
1 restraint	Δρ_min_ = −0.42 e Å^−^^3^

### Fractional atomic coordinates and isotropic or equivalent isotropic displacement parameters (Å^2^)

**Table:**

	*x*	*y*	*z*	*U*_iso_*/*U*_eq_	
V1	1.03271 (5)	0.59868 (4)	0.20412 (3)	0.01980 (12)	
O1	1.23385 (19)	0.48290 (15)	0.12970 (13)	0.0226 (3)	
O2	0.90127 (19)	0.68028 (15)	0.35967 (13)	0.0215 (3)	
O3	0.5685 (2)	0.44133 (16)	0.65797 (15)	0.0270 (4)	
H3	0.642 (3)	0.413 (3)	0.5925 (17)	0.052 (9)*	
O4	0.8957 (2)	0.58562 (17)	0.12891 (14)	0.0268 (4)	
O5	1.1345 (2)	0.75747 (15)	0.15366 (13)	0.0242 (3)	
N1	0.9533 (2)	0.43490 (19)	0.32837 (15)	0.0200 (4)	
N2	0.8269 (2)	0.46643 (18)	0.44290 (15)	0.0197 (4)	
C1	1.4016 (3)	0.3021 (2)	0.0049 (2)	0.0262 (5)	
H1A	1.5157	0.3390	0.0056	0.039*	
H1B	1.3764	0.3345	−0.0709	0.039*	
H1C	1.4138	0.2016	0.0077	0.039*	
C2	1.2480 (3)	0.3493 (2)	0.11473 (19)	0.0226 (5)	
C3	1.1325 (3)	0.2624 (2)	0.1970 (2)	0.0237 (5)	
H3a	1.1440	0.1681	0.1786	0.028*	
C4	0.9969 (3)	0.3040 (2)	0.30769 (19)	0.0211 (4)	
C5	0.9049 (3)	0.1972 (2)	0.4039 (2)	0.0246 (5)	
H5A	0.7716	0.2123	0.4263	0.037*	
H5B	0.9413	0.2040	0.4776	0.037*	
H5C	0.9418	0.1057	0.3704	0.037*	
C6	0.8042 (3)	0.5988 (2)	0.45013 (18)	0.0197 (4)	
C7	0.6691 (3)	0.6619 (2)	0.55786 (18)	0.0191 (4)	
C8	0.5524 (3)	0.5805 (2)	0.65770 (19)	0.0202 (4)	
C9	0.4232 (3)	0.6431 (2)	0.75423 (19)	0.0213 (4)	
H9	0.3474	0.5883	0.8199	0.026*	
C10	0.3994 (3)	0.7875 (2)	0.75869 (19)	0.0197 (4)	
C11	0.2617 (3)	0.8555 (2)	0.8555 (2)	0.0240 (5)	
H11	0.1839	0.8029	0.9221	0.029*	
C12	0.2394 (3)	0.9953 (2)	0.8542 (2)	0.0262 (5)	
H12	0.1459	1.0386	0.9197	0.031*	
C13	0.3529 (3)	1.0762 (2)	0.7572 (2)	0.0257 (5)	
H13	0.3356	1.1733	0.7572	0.031*	
C14	0.4881 (3)	1.0143 (2)	0.6631 (2)	0.0227 (5)	
H14	0.5653	1.0692	0.5982	0.027*	
C15	0.5148 (3)	0.8695 (2)	0.66085 (18)	0.0187 (4)	
C16	0.6479 (3)	0.8029 (2)	0.56254 (18)	0.0194 (4)	
H16	0.7259	0.8569	0.4972	0.023*	
C17	1.2231 (3)	0.8381 (2)	0.2134 (2)	0.0286 (5)	
H17A	1.1333	0.8741	0.2889	0.043*	
H17B	1.2789	0.9146	0.1577	0.043*	
H17C	1.3185	0.7813	0.2350	0.043*	

### Atomic displacement parameters (Å^2^)

**Table:**

	*U*^11^	*U*^22^	*U*^33^	*U*^12^	*U*^13^	*U*^23^
V1	0.01469 (19)	0.0299 (2)	0.01415 (19)	−0.00336 (14)	−0.00424 (14)	0.00440 (14)
O1	0.0149 (7)	0.0334 (8)	0.0186 (8)	−0.0038 (6)	−0.0041 (6)	0.0014 (6)
O2	0.0170 (7)	0.0285 (8)	0.0160 (7)	−0.0034 (6)	−0.0019 (6)	0.0047 (6)
O3	0.0288 (9)	0.0262 (8)	0.0201 (8)	−0.0047 (7)	0.0000 (7)	0.0030 (6)
O4	0.0197 (8)	0.0417 (9)	0.0203 (8)	−0.0049 (7)	−0.0089 (6)	0.0057 (7)
O5	0.0217 (8)	0.0306 (8)	0.0167 (8)	−0.0047 (6)	−0.0013 (6)	0.0030 (6)
N1	0.0127 (8)	0.0318 (10)	0.0147 (9)	−0.0022 (7)	−0.0037 (7)	0.0017 (7)
N2	0.0134 (8)	0.0310 (10)	0.0138 (8)	−0.0012 (7)	−0.0036 (7)	0.0019 (7)
C1	0.0187 (11)	0.0391 (13)	0.0203 (11)	−0.0008 (9)	−0.0058 (9)	−0.0006 (9)
C2	0.0183 (11)	0.0336 (12)	0.0185 (11)	−0.0018 (9)	−0.0096 (9)	−0.0002 (9)
C3	0.0213 (11)	0.0293 (11)	0.0218 (11)	−0.0009 (9)	−0.0083 (9)	−0.0029 (9)
C4	0.0165 (10)	0.0297 (11)	0.0200 (11)	−0.0032 (8)	−0.0100 (8)	0.0012 (9)
C5	0.0225 (11)	0.0292 (12)	0.0208 (11)	−0.0035 (9)	−0.0055 (9)	0.0021 (9)
C6	0.0140 (10)	0.0319 (12)	0.0146 (10)	−0.0035 (8)	−0.0072 (8)	0.0048 (8)
C7	0.0140 (10)	0.0296 (11)	0.0140 (10)	−0.0031 (8)	−0.0053 (8)	0.0044 (8)
C8	0.0183 (10)	0.0272 (11)	0.0175 (10)	−0.0042 (8)	−0.0095 (8)	0.0045 (8)
C9	0.0179 (10)	0.0305 (11)	0.0157 (10)	−0.0068 (9)	−0.0059 (8)	0.0066 (8)
C10	0.0157 (10)	0.0312 (11)	0.0148 (10)	−0.0045 (8)	−0.0085 (8)	0.0038 (8)
C11	0.0164 (10)	0.0373 (13)	0.0175 (11)	−0.0034 (9)	−0.0049 (8)	0.0043 (9)
C12	0.0192 (11)	0.0370 (13)	0.0206 (11)	0.0031 (9)	−0.0049 (9)	−0.0019 (9)
C13	0.0259 (12)	0.0280 (11)	0.0250 (12)	−0.0006 (9)	−0.0110 (9)	−0.0002 (9)
C14	0.0211 (11)	0.0293 (11)	0.0190 (11)	−0.0053 (9)	−0.0083 (9)	0.0061 (9)
C15	0.0153 (10)	0.0271 (11)	0.0157 (10)	−0.0022 (8)	−0.0082 (8)	0.0029 (8)
C16	0.0139 (10)	0.0300 (11)	0.0152 (10)	−0.0064 (8)	−0.0065 (8)	0.0073 (8)
C17	0.0321 (13)	0.0277 (12)	0.0227 (12)	−0.0055 (10)	−0.0032 (10)	−0.0023 (9)

### Geometric parameters (Å, °)

**Table:**

V1—O4	1.5850 (15)	C5—H5C	0.9800
V1—O5	1.7693 (15)	C6—C7	1.465 (3)
V1—O1	1.8504 (15)	C7—C16	1.378 (3)
V1—O2	1.9242 (15)	C7—C8	1.439 (3)
V1—N1	2.0632 (17)	C8—C9	1.365 (3)
O1—C2	1.319 (3)	C9—C10	1.412 (3)
O2—C6	1.322 (2)	C9—H9	0.9500
O3—C8	1.357 (3)	C10—C11	1.420 (3)
O3—H3	0.828 (10)	C10—C15	1.425 (3)
O5—C17	1.416 (3)	C11—C12	1.365 (3)
N1—C4	1.315 (3)	C11—H11	0.9500
N1—N2	1.390 (2)	C12—C13	1.408 (3)
N2—C6	1.297 (3)	C12—H12	0.9500
C1—C2	1.488 (3)	C13—C14	1.367 (3)
C1—H1A	0.9800	C13—H13	0.9500
C1—H1B	0.9800	C14—C15	1.417 (3)
C1—H1C	0.9800	C14—H14	0.9500
C2—C3	1.366 (3)	C15—C16	1.408 (3)
C3—C4	1.414 (3)	C16—H16	0.9500
C3—H3a	0.9500	C17—H17A	0.9800
C4—C5	1.504 (3)	C17—H17B	0.9800
C5—H5A	0.9800	C17—H17C	0.9800
C5—H5B	0.9800		
			
O4—V1—O5	105.62 (8)	N2—C6—O2	120.77 (19)
O4—V1—O1	105.47 (8)	N2—C6—C7	121.02 (18)
O5—V1—O1	98.56 (7)	O2—C6—C7	118.20 (18)
O4—V1—O2	108.97 (7)	C16—C7—C8	118.74 (19)
O5—V1—O2	87.78 (6)	C16—C7—C6	119.59 (18)
O1—V1—O2	141.78 (6)	C8—C7—C6	121.63 (19)
O4—V1—N1	98.68 (7)	O3—C8—C9	118.97 (19)
O5—V1—N1	153.82 (7)	O3—C8—C7	121.06 (19)
O1—V1—N1	83.93 (7)	C9—C8—C7	120.0 (2)
O2—V1—N1	75.08 (7)	C8—C9—C10	121.51 (19)
C2—O1—V1	131.01 (13)	C8—C9—H9	119.2
C6—O2—V1	117.27 (13)	C10—C9—H9	119.2
C8—O3—H3	112 (2)	C9—C10—C11	122.76 (19)
C17—O5—V1	128.78 (13)	C9—C10—C15	119.14 (19)
C4—N1—N2	116.69 (17)	C11—C10—C15	118.09 (19)
C4—N1—V1	127.25 (14)	C12—C11—C10	120.9 (2)
N2—N1—V1	115.79 (13)	C12—C11—H11	119.5
C6—N2—N1	108.95 (17)	C10—C11—H11	119.5
C2—C1—H1A	109.5	C11—C12—C13	121.0 (2)
C2—C1—H1B	109.5	C11—C12—H12	119.5
H1A—C1—H1B	109.5	C13—C12—H12	119.5
C2—C1—H1C	109.5	C14—C13—C12	119.7 (2)
H1A—C1—H1C	109.5	C14—C13—H13	120.1
H1B—C1—H1C	109.5	C12—C13—H13	120.1
O1—C2—C3	121.7 (2)	C13—C14—C15	120.9 (2)
O1—C2—C1	114.88 (19)	C13—C14—H14	119.5
C3—C2—C1	123.4 (2)	C15—C14—H14	119.5
C2—C3—C4	124.2 (2)	C16—C15—C14	122.15 (19)
C2—C3—H3a	117.9	C16—C15—C10	118.45 (19)
C4—C3—H3a	117.9	C14—C15—C10	119.37 (19)
N1—C4—C3	120.71 (19)	C7—C16—C15	122.19 (18)
N1—C4—C5	119.81 (19)	C7—C16—H16	118.9
C3—C4—C5	119.47 (19)	C15—C16—H16	118.9
C4—C5—H5A	109.5	O5—C17—H17A	109.5
C4—C5—H5B	109.5	O5—C17—H17B	109.5
H5A—C5—H5B	109.5	H17A—C17—H17B	109.5
C4—C5—H5C	109.5	O5—C17—H17C	109.5
H5A—C5—H5C	109.5	H17A—C17—H17C	109.5
H5B—C5—H5C	109.5	H17B—C17—H17C	109.5
			
O4—V1—O1—C2	−60.18 (18)	N1—N2—C6—O2	−3.3 (2)
O5—V1—O1—C2	−169.10 (17)	N1—N2—C6—C7	175.95 (16)
O2—V1—O1—C2	93.57 (19)	V1—O2—C6—N2	13.9 (2)
N1—V1—O1—C2	37.18 (17)	V1—O2—C6—C7	−165.34 (13)
O4—V1—O2—C6	81.17 (14)	N2—C6—C7—C16	−179.81 (18)
O5—V1—O2—C6	−173.06 (14)	O2—C6—C7—C16	−0.5 (3)
O1—V1—O2—C6	−72.03 (17)	N2—C6—C7—C8	−2.0 (3)
N1—V1—O2—C6	−13.05 (13)	O2—C6—C7—C8	177.25 (17)
O4—V1—O5—C17	159.55 (17)	C16—C7—C8—O3	−179.55 (18)
O1—V1—O5—C17	−91.66 (18)	C6—C7—C8—O3	2.6 (3)
O2—V1—O5—C17	50.46 (17)	C16—C7—C8—C9	0.4 (3)
N1—V1—O5—C17	2.0 (3)	C6—C7—C8—C9	−177.42 (18)
O4—V1—N1—C4	77.84 (18)	O3—C8—C9—C10	−179.64 (18)
O5—V1—N1—C4	−124.0 (2)	C7—C8—C9—C10	0.4 (3)
O1—V1—N1—C4	−26.94 (17)	C8—C9—C10—C11	177.58 (19)
O2—V1—N1—C4	−174.72 (18)	C8—C9—C10—C15	−0.8 (3)
O4—V1—N1—N2	−95.99 (14)	C9—C10—C11—C12	−177.81 (19)
O5—V1—N1—N2	62.2 (2)	C15—C10—C11—C12	0.6 (3)
O1—V1—N1—N2	159.23 (13)	C10—C11—C12—C13	−0.3 (3)
O2—V1—N1—N2	11.45 (12)	C11—C12—C13—C14	−0.3 (3)
C4—N1—N2—C6	177.68 (17)	C12—C13—C14—C15	0.7 (3)
V1—N1—N2—C6	−7.82 (19)	C13—C14—C15—C16	177.35 (19)
V1—O1—C2—C3	−29.6 (3)	C13—C14—C15—C10	−0.4 (3)
V1—O1—C2—C1	152.33 (15)	C9—C10—C15—C16	0.4 (3)
O1—C2—C3—C4	−4.0 (3)	C11—C10—C15—C16	−178.07 (18)
C1—C2—C3—C4	173.90 (19)	C9—C10—C15—C14	178.22 (18)
N2—N1—C4—C3	−176.11 (17)	C11—C10—C15—C14	−0.3 (3)
V1—N1—C4—C3	10.1 (3)	C8—C7—C16—C15	−0.8 (3)
N2—N1—C4—C5	2.5 (3)	C6—C7—C16—C15	177.05 (18)
V1—N1—C4—C5	−171.27 (14)	C14—C15—C16—C7	−177.34 (19)
C2—C3—C4—N1	11.5 (3)	C10—C15—C16—C7	0.4 (3)
C2—C3—C4—C5	−167.2 (2)		

### Hydrogen-bond geometry (Å, °)

**Table:**

*D*—H···*A*	*D*—H	H···*A*	*D*···*A*	*D*—H···*A*
O3—H3···N2	0.83 (1)	1.91 (2)	2.623 (2)	144 (3)
